# 2-Chloro-4-hy­droxy­anilinium chloride

**DOI:** 10.1107/S2414314625008934

**Published:** 2025-10-17

**Authors:** Sindhu V. Bai, Patrick F. Mensah, Guoqiang Li, Frank R. Fronczek, Rao M. Uppu

**Affiliations:** a Department of Environmental Toxicology Southern University and A&M College Baton Rouge Louisiana 70813 USA; b Department of Mechanical Engineering Southern University and A&M College Baton Rouge Louisiana 70813 USA; c Department of Mechanical and Industrial Engineering Louisiana State University,Baton Rouge Louisiana 70803 USA; d Department of Chemistry Louisiana State University,Baton Rouge Louisiana 70803 USA

**Keywords:** crystal structure, 4-amino-3-chloro­aniline, lenvatinib and tivozanib, tyrosine kinase inhibitors, anti­cancer drugs and inter­mediates

## Abstract

The title compound, C_6_H_7_ClNO^+^·Cl^−^, crystallizes in ortho­rhom­bic space group *Pnma* with both cations and anions lying on a mirror plane in the crystal. The C—Cl distance is 1.7289 (6) Å, the C—N distance is 1.4590 (8) Å, and the C—O distance is 1.3617 (8) Å. Parallel mol­ecules form stacks with inter­planar spacing 3.1473 (2) Å, but slipped by 1.97 Å. The NH_3_^+^ substituent donates three inter­molecular hydrogen bonds to chloride ions having N⋯Cl distances in the range 3.1514 (6)–3.3019 (2) Å, and the OH group donates an inter­molecular hydrogen bond to chloride with O⋯Cl distance 3.0671 (6) Å. The chloro substituent and OH group do not accept hydrogen bonds from NH or OH.

## Abstract 2

The title compound crystallizes in ortho­rhom­bic space group *Pnma* with both cations and anions lying on a mirror plane in the crystal. Parallel mol­ecules form stacks with inter­planar spacing 3.1473 (2) Å, but slipped by 1.97 Å.

## Graphical abstract

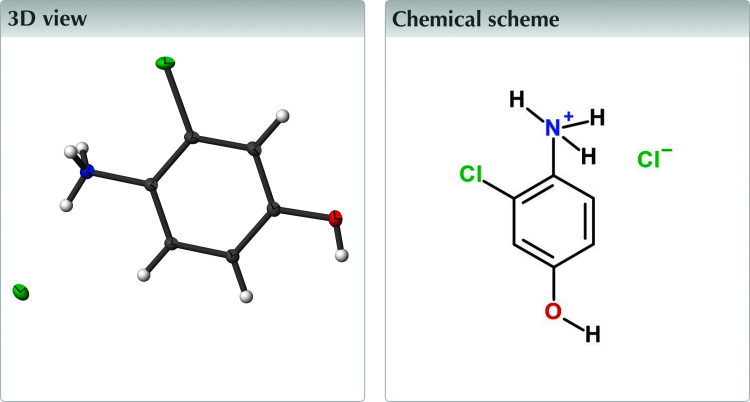

## Structure description

4-Hy­droxy-2-chloro­anilinium chloride is the hydro­chloride salt form of the well-established inter­mediate 4-amino-3-chloro­phenol. This compound occupies a central role in pharmaceutical chemistry as a key starting material in the large-scale synthesis of the multikinase inhibitors lenvatinib and tivozanib, which act primarily through potent inhibition of vascular endothelial growth factor receptor (VEGFR) signaling pathways (CN 104326924 A, 2015 ▸; EP 3620452A1, 2020 ▸; Nair *et al.*, 2015 ▸). The simultaneous presence of an aniline amino and a phenolic hydroxyl group renders this scaffold a privileged synthon, particularly suited for the preparation of phen­oxy-anilide derivatives. Such derivatives have been widely adopted in kinase inhibitor design and continue to attract attention as versatile structural motifs for next generation anti­cancer therapeutics (Kumar *et al.*, 2015 ▸).

Structural biology investigations have demonstrated that the phen­oxy-anilide fragment derived from this inter­mediate is a critical determinant of VEGFR binding. In lenvatinib, this fragment stabilizes occupancy within the ATP-binding site and an adjacent hydro­phobic pocket of VEGFR2 (Pan *et al.*, 2021 ▸; Okamoto *et al.*, 2014 ▸; Yamamoto *et al.*, 2014 ▸), thereby enabling potent inhibition across VEGFR1–3 as well as FGFR1–4. Tivozanib employs the same pharmacophore unit to achieve selective kinase blockade (CN 104326924A, 2015 ▸). Beyond these clinical examples, the scaffold continues to be featured in discovery libraries and in the design of dual-target inhibitors. Recent advances have further enhanced its industrial relevance, with continuous-flow synthetic routes improving both safety and efficiency (CN 107739313A, 2020 ▸).

Against this background, it was considered that the crystal structure of 4-amino-3-chloro­phenol hydro­chloride would provide valuable insights into its mol­ecular conformation, hydrogen-bonding patterns, and supra­molecular packing. These structural parameters are expected not only to clarify the stability of the salt form, but also to rationalize its synthetic utility in downstream coupling reactions. In view of its broad applications to mechanistic understanding and its potential for new drug development, we therefore undertook an X-ray diffraction study of the title compound at 100 K.

**Figure 1:**
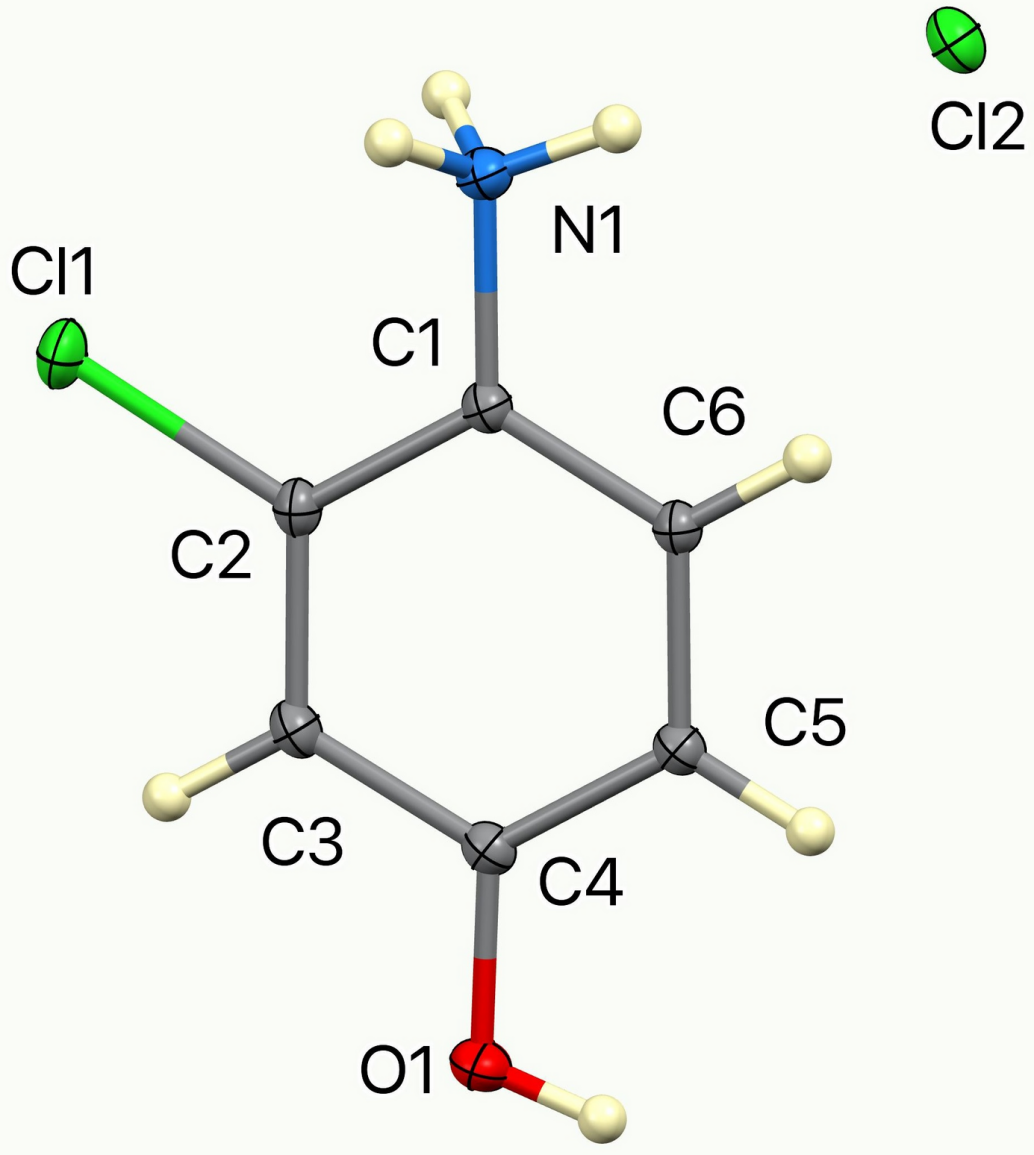
The asymmetric unit of the title compound with 50% displacement ellipsoids.

**Figure 2:**
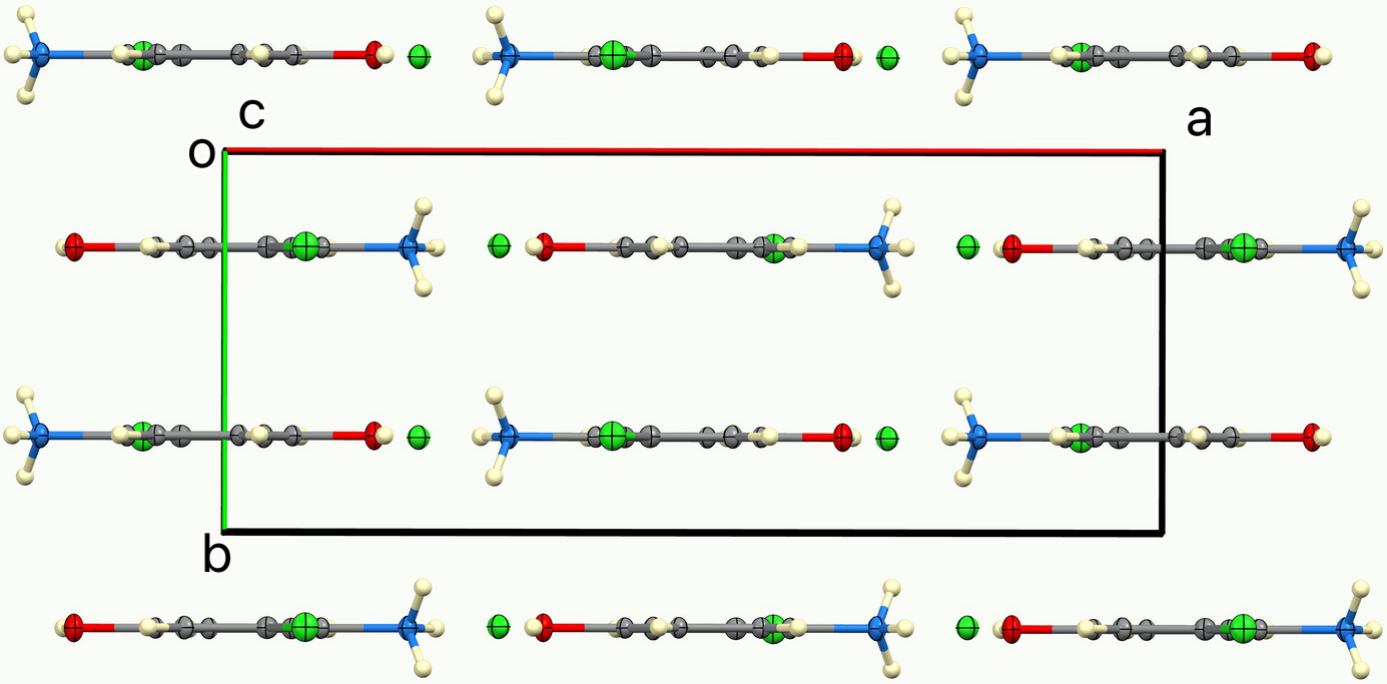
A view of the unit-cell contents, in projection down the *c* axis.

**Figure 3:**
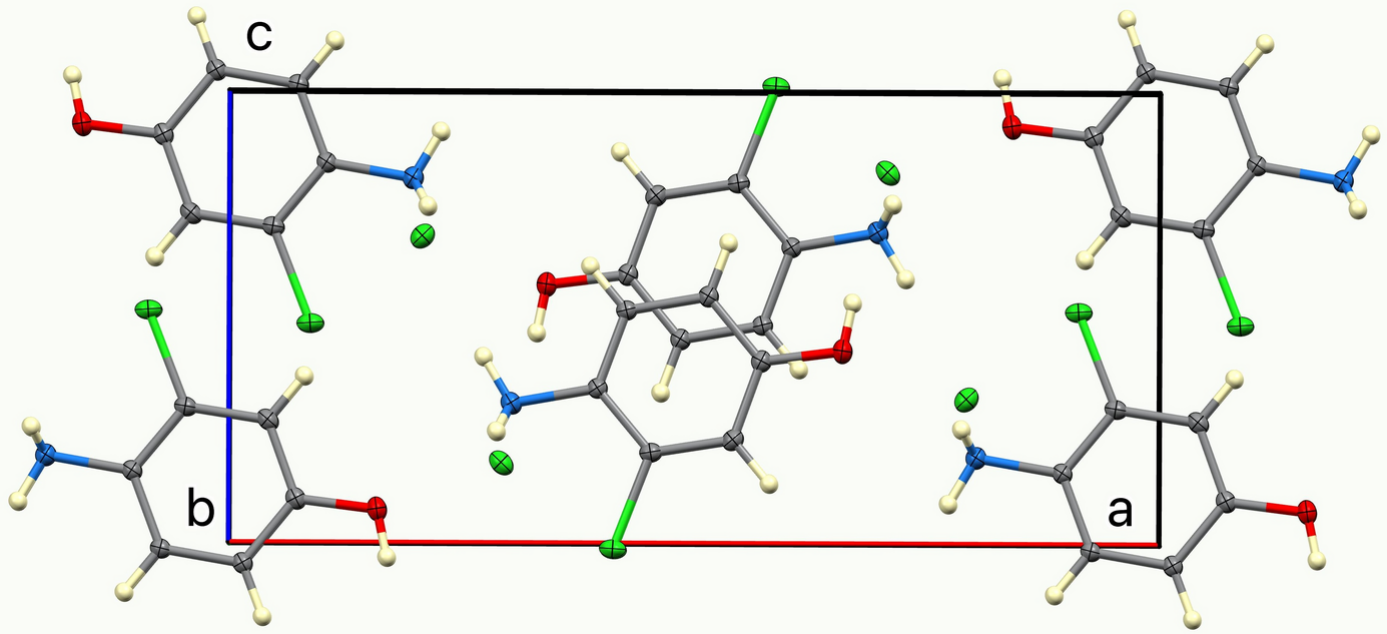
A view of the unit-cell contents, in projection down the *b* axis.

The ellipsoids and atom numbering are shown in Fig. 1 ▸. Both cations and anions lie on a mirror plane, thus, except for two ammonium H atoms related by the mirror, the cation is rigorously planar, and all cations are parallel. This is shown in Fig. 2 ▸, a view of the unit cell down the *c* axis. The inter­planar spacing is half the *b* axial length, 3.1473 (2) Å, but cations related by an inversion center are horizontally slipped by 1.97 Å, as shown in the view down the *b* axis, Fig. 3 ▸.

**Figure 4:**
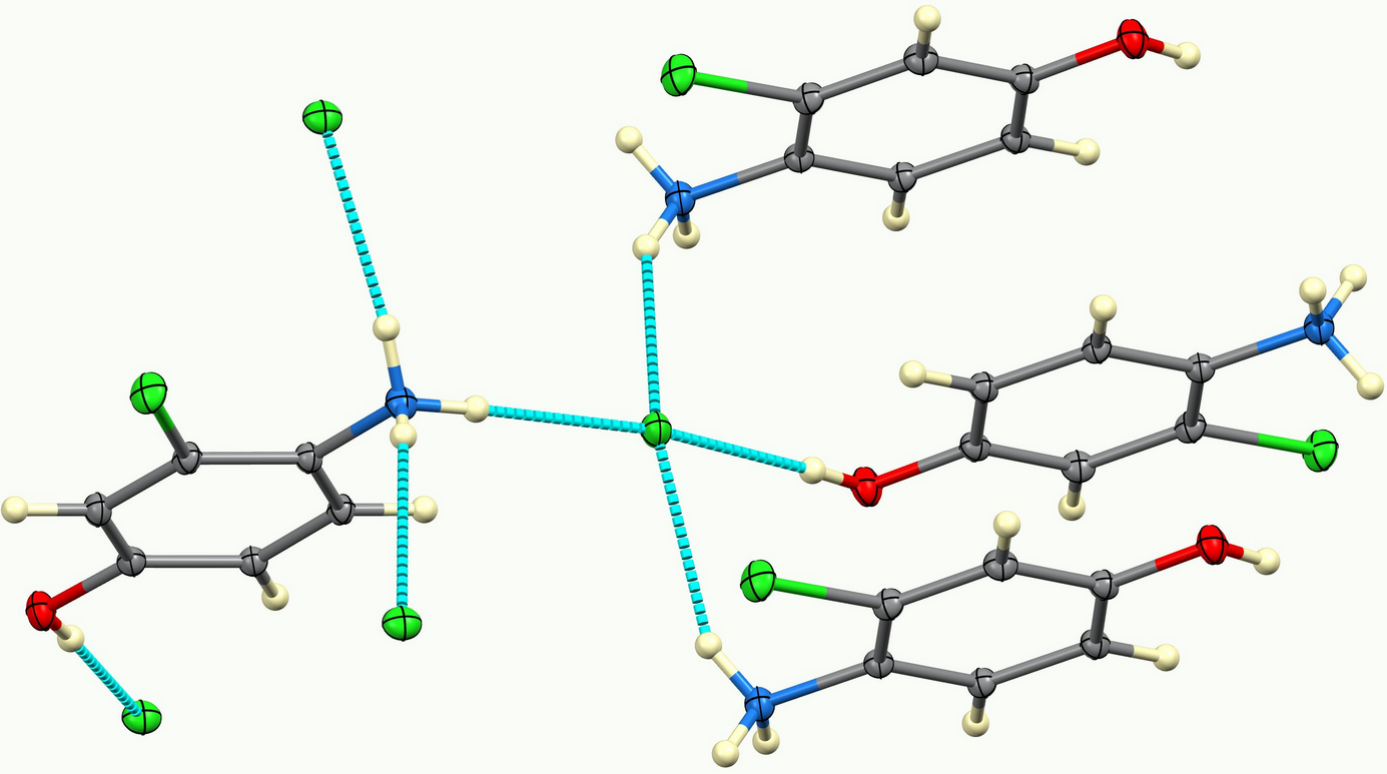
Hydrogen bonding shown as dashed bonds.

Hydrogen bonding from both the NH_3_^+^ and OH donors involve chloride only as the acceptor, not the chloro substituent nor the OH group. As shown in Fig. 4 ▸, the ammonium group donates to three different chloride ions, and the OH group donates to a fourth. Thus, chloride accepts four hydrogen bonds from NH and OH. There are longer C—H⋯Cl inter­actions to both chloride and the chloro substituent, as detailed in Table 1 ▸.

## Synthesis and crystallization

4-Hy­droxy-2-chloro­anilinium chloride (CAS 52671–64-4; purity: 98%) was obtained from AmBeed, Buffalo Grove, IL and was used without further purification. Crystallization was performed in ethanol by slow cooling of a hot, nearly saturated solution. The sample was clarified by filtration using Whatman #1 filter paper. Single crystals of the title compound were colorless needles.

## Refinement

Crystal data, data collection and structure refinement details are summarized in Table 2 ▸. Three reflections were omitted because of beamstop problems.

## Supplementary Material

Crystal structure: contains datablock(s) I. DOI: 10.1107/S2414314625008934/tk4119sup1.cif

Structure factors: contains datablock(s) I. DOI: 10.1107/S2414314625008934/tk4119Isup2.hkl

Supporting information file. DOI: 10.1107/S2414314625008934/tk4119Isup3.cml

CCDC reference: 2495195

Additional supporting information:  crystallographic information; 3D view; checkCIF report

## Figures and Tables

**Table 1:** Hydrogen-bond geometry (Å, °)

*D*—H⋯*A*	*D*—H	H⋯*A*	*D*⋯*A*	*D*—H⋯*A*
O1—H1⋯Cl2^i^	0.82 (1)	2.25 (1)	3.0671 (6)	177 (2)
N1—H1*N*⋯Cl2^ii^	0.856 (12)	2.531 (13)	3.3019 (2)	150.4 (10)
N1—H2*N*⋯Cl2	0.90 (2)	2.25 (2)	3.1514 (6)	176 (1)
C5—H5⋯Cl2^i^	0.95	2.90	3.6290 (6)	134
C6—H6⋯Cl1^iii^	0.95	2.84	3.5519 (6)	132
C6—H6⋯Cl2	0.95	2.84	3.6195 (6)	140
C6—H6⋯Cl1^iii^	0.95	2.84	3.5519 (6)	132

Symmetry codes: (i) 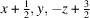; (ii) 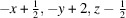; (iii) 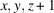.

**Table 2:** Experimental details

Crystal data	
Chemical formula	C_6_H_7_ClNO^+^·Cl^−^
*M*_r_	180.03
Crystal system, space group	Orthorhombic, *Pnma*
Temperature (K)	100
*a*, *b*, *c* (Å)	15.5044 (4), 6.2945 (2), 7.5260 (2)
*V* (Å^3^)	734.48 (4)
*Z*	4
Radiation type	Ag *K*α, λ = 0.56086 Å
μ (mm^−1^)	0.41
Crystal size (mm)	0.28 × 0.20 × 0.15

Data collection	
Diffractometer	Bruker D8 Venture DUO with Photon III C14
Absorption correction	Multi-scan (*SADABS*; Krause *et al.*, 2015 ▸)
*T*_min_, *T*_max_	0.881, 0.940
No. of measured, independent and observed [*I* > 2σ(*I*)] reflections	41806, 3668, 3193
*R*_int_	0.053
(sin θ/λ)_max_ (Å^−1^)	1.042

Refinement	
*R*[*F*^2^ > 2σ(*F*^2^)], *wR*(*F*^2^), *S*	0.031, 0.089, 1.05
No. of reflections	3668
No. of parameters	68
No. of restraints	1
H-atom treatment	H atoms treated by a mixture of independent and constrained refinement
Δρ_max_, Δρ_min_ (e Å^−3^)	0.98, −0.50

Computer programs: *APEX5* and *SAINT* (Bruker, 2016 ▸), *SHELXT2018/2* (Sheldrick, 2015 ▸a), *SHELXL2019/1* (Sheldrick, 2015 ▸b), *Mercury* (Macrae *et al.*, 2020 ▸) and *publCIF* (Westrip, 2010 ▸).

## full crystallographic data

### 2-Chloro-4-hydroxyanilinium chloride Crystal data

**Table:**

C_6_H_7_ClNO^+^·Cl^−^	*D*_x_ = 1.628 Mg m^−^^3^
*M**_r_* = 180.03	Ag *K*α radiation, λ = 0.56086 Å
Orthorhombic, *P**n**m**a*	Cell parameters from 9968 reflections
*a* = 15.5044 (4) Å	θ = 3.0–35.2°
*b* = 6.2945 (2) Å	µ = 0.41 mm^−^^1^
*c* = 7.5260 (2) Å	*T* = 100 K
*V* = 734.48 (4) Å^3^	Needle fragment, colourless
*Z* = 4	0.28 × 0.20 × 0.15 mm
*F*(000) = 368	

### 2-Chloro-4-hydroxyanilinium chloride Data collection

**Table:**

Bruker D8 Venture DUO with Photon III C14 diffractometer	3193 reflections with *I* > 2σ(*I*)
Radiation source: IµS 3.0 microfocus	*R*_int_ = 0.053
φ and ω scans	θ_max_ = 35.8°, θ_min_ = 3.0°
Absorption correction: multi-scan (SADABS; Krause *et al.*, 2015)	*h* = −32→32
*T*_min_ = 0.881, *T*_max_ = 0.940	*k* = −13→11
41806 measured reflections	*l* = −14→15
3668 independent reflections	

### 2-Chloro-4-hydroxyanilinium chloride Refinement

**Table:**

Refinement on *F*^2^	Primary atom site location: structure-invariant direct methods
Least-squares matrix: full	Secondary atom site location: difference Fourier map
*R*[*F*^2^ > 2σ(*F*^2^)] = 0.031	Hydrogen site location: mixed
*wR*(*F*^2^) = 0.089	H atoms treated by a mixture of independent and constrained refinement
*S* = 1.05	*w* = 1/[σ^2^(*F*_o_^2^) + (0.0489*P*)^2^ + 0.0955*P*] where *P* = (*F*_o_^2^ + 2*F*_c_^2^)/3
3668 reflections	(Δ/σ)_max_ = 0.001
68 parameters	Δρ_max_ = 0.98 e Å^−^^3^
1 restraint	Δρ_min_ = −0.50 e Å^−^^3^

### 2-Chloro-4-hydroxyanilinium chloride Special details

**Table:**

Geometry. All esds (except the esd in the dihedral angle between two l.s. planes) are estimated using the full covariance matrix. The cell esds are taken into account individually in the estimation of esds in distances, angles and torsion angles; correlations between esds in cell parameters are only used when they are defined by crystal symmetry. An approximate (isotropic) treatment of cell esds is used for estimating esds involving l.s. planes.
Refinement. All H atoms were located in difference maps and those on C were thereafter treated as riding in geometrically idealized positions with C—H distances of 0.95 Å. The coordinates of the N—H and O—H hydrogen atoms were refined, with the O—H distance restrained to 0.85 Å. *U*_iso_(H) values were assigned as 1.2*U*_eq_ for the attached atom (1.5 for NH_3_ and OH).

### 2-Chloro-4-hydroxyanilinium chloride Fractional atomic coordinates and isotropic or equivalent isotropic displacement parameters (Å^2^)

**Table:**

	*x*	*y*	*z*	*U*_iso_*/*U*_eq_	
Cl1	0.41328 (2)	0.750000	−0.01348 (2)	0.01726 (4)	
O1	0.65937 (3)	0.750000	0.42625 (8)	0.01557 (9)	
H1	0.6701 (10)	0.750000	0.5329 (19)	0.023*	
N1	0.30340 (4)	0.750000	0.30926 (8)	0.01320 (8)	
H1N	0.2875 (7)	0.857 (2)	0.2467 (14)	0.020*	
H2N	0.2735 (10)	0.750000	0.412 (2)	0.020*	
C1	0.39608 (4)	0.750000	0.34282 (8)	0.01067 (8)	
C2	0.45356 (4)	0.750000	0.20074 (8)	0.01172 (8)	
C3	0.54184 (4)	0.750000	0.22936 (8)	0.01262 (9)	
H3	0.580887	0.750000	0.132087	0.015*	
C4	0.57223 (4)	0.750000	0.40361 (8)	0.01128 (8)	
C5	0.51515 (4)	0.750000	0.54682 (8)	0.01106 (8)	
H5	0.536501	0.750000	0.665132	0.013*	
C6	0.42714 (4)	0.750000	0.51543 (8)	0.01064 (8)	
H6	0.387967	0.750000	0.612496	0.013*	
Cl2	0.20751 (2)	0.750000	0.67846 (2)	0.01519 (4)	

### 2-Chloro-4-hydroxyanilinium chloride Atomic displacement parameters (Å^2^)

**Table:**

	*U*^11^	*U*^22^	*U*^33^	*U*^12^	*U*^13^	*U*^23^
Cl1	0.01969 (8)	0.02336 (9)	0.00874 (6)	0.000	−0.00069 (4)	0.000
O1	0.00992 (15)	0.0218 (2)	0.01502 (19)	0.000	0.00072 (13)	0.000
N1	0.01106 (17)	0.0165 (2)	0.01204 (18)	0.000	−0.00098 (14)	0.000
C1	0.01023 (17)	0.0120 (2)	0.00982 (18)	0.000	0.00045 (14)	0.000
C2	0.0131 (2)	0.0133 (2)	0.00879 (17)	0.000	0.00073 (15)	0.000
C3	0.0121 (2)	0.0145 (2)	0.01128 (19)	0.000	0.00239 (15)	0.000
C4	0.01043 (17)	0.01161 (19)	0.0118 (2)	0.000	0.00095 (14)	0.000
C5	0.01029 (17)	0.0120 (2)	0.01085 (18)	0.000	0.00049 (14)	0.000
C6	0.01086 (18)	0.0119 (2)	0.00919 (18)	0.000	0.00044 (13)	0.000
Cl2	0.01500 (7)	0.01586 (7)	0.01472 (7)	0.000	0.00405 (4)	0.000

### 2-Chloro-4-hydroxyanilinium chloride Geometric parameters (Å, º)

**Table:**

Cl1—C2	1.7289 (6)	C1—C2	1.3919 (8)
O1—C4	1.3617 (8)	C2—C3	1.3856 (9)
O1—H1	0.820 (14)	C3—C4	1.3935 (9)
N1—C1	1.4590 (8)	C3—H3	0.9500
N1—H1N	0.856 (12)	C4—C5	1.3946 (8)
N1—H2N	0.899 (15)	C5—C6	1.3848 (8)
N1—H1N^i^	0.856 (12)	C5—H5	0.9500
C1—C6	1.3854 (8)	C6—H6	0.9500
			
C4—O1—H1	108.8 (12)	C2—C3—C4	118.71 (6)
C1—N1—H1N	112.3 (7)	C2—C3—H3	120.6
C1—N1—H2N	111.1 (10)	C4—C3—H3	120.6
H1N—N1—H2N	108.8 (9)	O1—C4—C3	116.96 (6)
C1—N1—H1N^i^	112.3 (7)	O1—C4—C5	122.20 (6)
H1N—N1—H1N^i^	103.2 (15)	C3—C4—C5	120.84 (6)
H2N—N1—H1N^i^	108.8 (9)	C6—C5—C4	119.57 (6)
C6—C1—C2	119.86 (6)	C6—C5—H5	120.2
C6—C1—N1	120.31 (5)	C4—C5—H5	120.2
C2—C1—N1	119.84 (5)	C5—C6—C1	120.16 (5)
C3—C2—C1	120.86 (6)	C5—C6—H6	119.9
C3—C2—Cl1	120.12 (5)	C1—C6—H6	119.9
C1—C2—Cl1	119.02 (5)		

Symmetry code: (i) *x*, −*y*+3/2, *z*.

### 2-Chloro-4-hydroxyanilinium chloride Hydrogen-bond geometry (Å, º)

**Table:**

*D*—H···*A*	*D*—H	H···*A*	*D*···*A*	*D*—H···*A*
O1—H1···Cl2^ii^	0.82 (1)	2.25 (1)	3.0671 (6)	177 (2)
N1—H1*N*···Cl2^iii^	0.856 (12)	2.531 (13)	3.3019 (2)	150.4 (10)
N1—H2*N*···Cl2	0.90 (2)	2.25 (2)	3.1514 (6)	176 (1)
C5—H5···Cl2^ii^	0.95	2.90	3.6290 (6)	134
C6—H6···Cl1^iv^	0.95	2.84	3.5519 (6)	132
C6—H6···Cl2	0.95	2.84	3.6195 (6)	140
C6—H6···Cl1^iv^	0.95	2.84	3.5519 (6)	132

Symmetry codes: (ii) *x*+1/2, *y*, −*z*+3/2; (iii) −*x*+1/2, −*y*+2, *z*−1/2; (iv) *x*, *y*, *z*+1.
